# Lower ^123^I-FP-CIT binding to the striatal dopamine transporter, but not to the extrastriatal serotonin transporter, in Parkinson's disease compared with dementia with Lewy bodies

**DOI:** 10.1016/j.nicl.2018.04.009

**Published:** 2018-04-06

**Authors:** Merijn Joling, Chris Vriend, Jessica J. van der Zande, Afina W. Lemstra, Odile A. van den Heuvel, Jan Booij, Henk W. Berendse

**Affiliations:** a Department of Neurology, VU University Medical Center, Amsterdam, The Netherlands; b Department of Radiology and Nuclear Medicine, Academic Medical Center, Amsterdam, The Netherlands; c Amsterdam Neuroscience, Amsterdam, The Netherlands; d Department of Psychiatry, VU University Medical Center, Amsterdam, The Netherlands; e Department of Anatomy and Neurosciences, VU University Medical Center, Amsterdam, The Netherlands; f Alzheimer Center, VU University Medical Center, Amsterdam, The Netherlands

**Keywords:** Parkinson's disease, Dementia with Lewy bodies, ^123^I-FP-CIT, SERT, DAT

## Abstract

In this retrospective cross-sectional study we compared ^123^I‑*N*‑ω‑fluoropropyl‑2β‑carbomethoxy‑3β‑(4‑iodophenyl)nortropane (^123^I-FP-CIT) binding to the striatal dopamine and the extrastriatal serotonin transporter (DAT and SERT, respectively) between Parkinson's disease (PD) and dementia with Lewy bodies (DLB) to gain more insight in the pathophysiology of the two diseases.

We compared ^123^I-FP-CIT single photon emission computed tomography scans of, age-, gender matched patients with cognitive decline in same range of severity with PD (n = 53) or DLB (n = 53) using a regions of interest (ROIs) approach. We derived ROIs anatomically from individual magnetic resonance imaging brain scans. To corroborate the ROI findings, we performed additional whole-brain voxel-based analyses.

In both ROI and voxel-based analyses, ^123^I-FP-CIT binding in PD patients was significantly lower in the bilateral posterior putamen than in DLB patients (left: *F*(1,103) = 18.363, *P* < 0.001, *ω*^*2*^ = 0.14; right: *F*(1,103) = 20.434, *P* < 0.001, *ω*^*2*^ = 0.15) (*P*_*corr*_ < 0.033). Caudate/putamen ratios were also significantly lower in DLB than in PD (U(105) = 724.0, *P* < 0.001). Extrastriatal SERT binding showed no difference between PD and DLB.

These results suggest similar involvement of serotonergic structures in the degenerative process in PD and DLB.

## Highlights

• This study is the first to extensively compare extrastriatal ^123^I-FP-CIT binding in PD and DLB.
• ^123^I-FP-CIT binding is lower in the posterior putamen of PD than DLB patients.
• There is no difference in extrastriatal ^123^I-FP-CIT binding between PD and DLB.

## 1 Introduction

Parkinson's disease (PD) and dementia with Lewy bodies (DLB) are both characterised by dopaminergic neurodegeneration and Lewy body pathology in the brain, mainly in the substantia nigra (Bethlem and Den Hartog Jager, 1960; Braak et al., 2003; Hansen et al., 1990). The dopaminergic neurodegeneration is associated with the classical motor symptoms of parkinsonism, which includes bradykinesia, rigidity, resting tremor and postural instability. Both PD and DLB also encompass non-motor symptoms such as depression, anxiety, hallucinations and cognitive decline. The clinical distinction between PD and DLB is currently based on the timing of the onset of cognitive decline relative to the onset of motor symptoms: DLB is diagnosed when cognitive decline appears before, or no longer than one year *after* the development of parkinsonism (one year rule) (McKeith et al., 2005), while PD is diagnosed when parkinsonism predates cognitive decline for more than a year. PD and DLB are thought to be manifestations of a single Lewy body-disease spectrum. However, we still do not know the extent of this spectrum, and why some patients have a PD phenotype rather than a DLB phenotype, and vice versa. It is therefore interesting to explore differences and similarities of both diseases.

Considering the clinical differences between PD and DLB in phenotype and disease course, one might expect a differential involvement of neurotransmitters systems. The results of neuropathological and molecular imaging suggest that the pattern of neurodegeneration in dopaminergic (Piggott et al., 1999), serotonergic (Roselli et al., 2010) and cholinergic (Hepp et al., 2013) systems differs between DLB and PD. In vivo it is possible to visualise both dopaminergic and serotoninergic systems with a single tracer, ^123^I‑*N*‑ω‑fluoropropyl‑2β‑carbomethoxy‑3β‑(4‑iodophenyl)nortropane (^123^I-FP-CIT). This well-validated single photon emission computed tomography (SPECT) radiotracer has high affinity for the presynaptic dopamine transporter (DAT) (Booij et al., 1997), and, additionally a modest affinity for the presynaptic serotonin transporter (SERT) (Abi-Dargham et al., 1996). Previous studies have shown that ^123^I-FP-CIT SPECT imaging is sensitive enough to study the integrity of the dopaminergic system in the striatum (Booij et al., 1999), and the serotoninergic system in extrastriatal brain areas (Koopman et al., 2012; Ziebell et al., 2010).

Striatal DAT loss in both PD and DLB has been well documented using ^123^I-FP-CIT SPECT. The reported differences between PD and DLB include a more extensive loss of DAT binding in the putamen than in the caudate nucleus in PD, which is also reflected in a flatter rostrocaudal (caudate-putamen) gradient in DLB than in PD (O'Brien et al., 2004; Walker et al., 2004). In addition to the differences in striatal ^123^I-FP-CIT binding, in a preliminary study, Roselli and co-workers reported lower extrastriatal ^123^I-FP-CIT binding to SERT in the midbrain in DLB (n = 16) than in PD patients (n = 15) (Roselli et al., 2010). In clinical studies the prevalence of neuropsychiatric symptoms associated with a serotonergic deficit, such as anxiety, appears to be different in DLB than in PD, although the results vary (Chiu et al., 2016; Kao et al., 2009). This observation is relevant, from both a scientific and a clinical point of view, because if we would be able to confirm the differences in the constellation of serotonergic degeneration in PD and DLB, this would stimulate research on the relationship between serotonergic degeneration and clinical symptoms in DLB.

In this cross-sectional molecular imaging study we aimed to obtain more information on possible DAT and SERT differences between PD and DLB. In line with the literature, we expected to find a difference in the rostrocaudal pattern of ^123^I-FP-CIT DAT binding between PD and DLB patients. In addition, we hypothesised that DLB patients would show a different pattern of ^123^I-FP-CIT binding in SERT-rich extrastriatal regions than PD patients.

## 2 Patients and methods

### 2.1 Participants

**Fig. 1:**
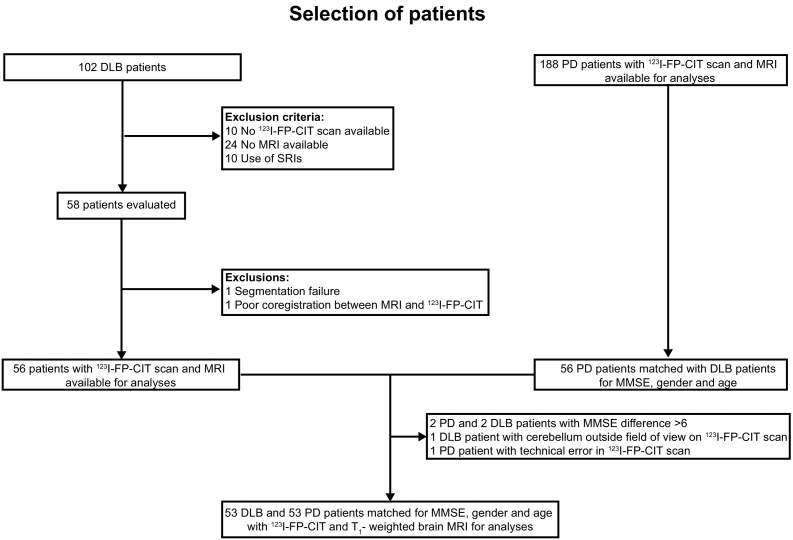
Patients included in the study - PD, Parkinson's disease; DLB, Dementia with Lewy bodies; MRI, magnetic resonance imaging; SRI, serotonin reuptake inhibitor; MMSE, mini mental state examination.

In this retrospective cross-sectional study we selected clinically diagnosed PD and DLB patients from consecutive cases that presented between December 2006 and March 2017 from both the outpatient clinic for movement disorders and the memory clinic (Amsterdam Dementia Cohort, Alzheimer Center (van der Flier et al., 2014)), both at the department of Neurology of the VU University Medical Center (VUmc) in Amsterdam, The Netherlands. We included probable DLB and PD patients in whom ^123^I-FP-CIT SPECT imaging had been performed, and a T_1_-weighted magnetic resonance imaging (MRI) brain scan and a mini mental state examination (MMSE) were available. PD and DLB patients on serotonin reuptake inhibitors (SRIs) were excluded, because these drugs may influence ^123^I-FP-CIT SERT binding (Booij et al., 2007). Apart from this, the use of common anti-parkinsonian drugs like levodopa, as well as dopamine agonists was not used as an exclusion criterion. From this selection, fewer DLB than PD patients were available for analysis, therefore we matched the DLB patients subject by subject with eligible PD patients based on age and gender, and MMSE scores in the same range of severity, since previous studies showed effects of ageing, gender and cognitive deficits on striatal ^123^I-FP-CIT binding (Siepel et al., 2014; Varrone et al., 2013). In- and exclusion criteria are listed in the flowchart in Fig. 1.

PD patients were diagnosed by a movement disorder specialist using the clinical diagnostic criteria of the United Kingdom PD Society Brain Bank criteria (Hughes et al., 1992). Severity of the motor symptoms was rated with the Unified Parkinson's Disease Rating Scale–motor section (UPDRS-III) (Fahn and Elton, 1987). The DLB patients were diagnosed in a multi-disciplinary meeting at the Alzheimer Center according to the McKeith criteria (McKeith et al., 2005). For these patients, the presence of motor symptoms (bradykinesia, rigidity, tremor, postural instability) was registered dichotomously. All included patients gave written informed consent to use their clinical and neuroimaging data for scientific purposes, and this procedure was approved by the local medical ethics committee.

### 2.2 ^123^I-FP-CIT SPECT–image acquisition and pre-processing

^123^I-FP-CIT was intravenously administered in a dose of approximately 185 MBq (specific activity >185 MBq/nmol; radiochemical purity >99%; produced as DaTSCAN™ according to good-manufacturing-practices criteria at GE Healthcare, Eindhoven, The Netherlands). Starting from 3, and finishing before 4 h after injection, static images were obtained for 30 min using a dual-head gamma camera (E.Cam; Siemens, Munich, Germany) with a fan-beam collimator. The images were reconstructed as described earlier (Vriend et al., 2014), and subsequently reoriented to the anterior-posterior commissure plane in Statistical Parametric Mapping 12 software (SPM 12; Wellcome Trust Centre for Neuroimaging, London, UK).

### 2.3 MRI T_1_–image acquisition

Structural 3D T_1_-weighted images, used for individually tailored segmentation of subcortical structures and co-registration, were acquired on nine different MRI systems at the VUmc (Amsterdam, The Netherlands).

### 2.4 Region-of-interest atlas-based construction

Regions of interest (ROIs) included the bilateral striatal nucleus accumbens, anterior caudate nucleus and anterior and posterior putamen; and the bilateral extrastriatal amygdala, hippocampus and thalamus. We used FreeSurfer 6.0 (Athinoula A. Martinos Center for Biomedical Imaging, Boston, MA, USA) with default settings on the T_1_-weighted MRI scans to individually segment the ROIs. The putamen and caudate nucleus were divided into an anterior and posterior part by a line perpendicular to the anterior commissure. To avoid spill-over effects when calculating the DAT binding ratios, we combined the anterior caudate nucleus and the nucleus accumbens FreeSurfer segmentations into one ROI (left and right separately). All ROIs were visually inspected for segmentation errors.

### 2.5 ^123^I-FP-CIT SPECT and T_1_ co-registration

We co-registered the ^123^I-FP-CIT SPECT scans with the T_1_-weighted MRI scans with SPM 12 by using a previously established method (Joling et al., 2018) in which T_1_-weighted images with superimposed hyper-intense FreeSurfer segmentations of the striatal regions (caudate nucleus, putamen and nucleus accumbens) were used to create a common landmark in MRI and SPECT to optimise co-registration.

### 2.6 ^123^I-FP-CIT SPECT–image analysis

We calculated binding ratios with the cerebellum as the reference region (REF; WFU Pickatlas, Wake Forest University, Winston-Salem, NC, USA; automated anatomical labelling atlas; bilateral Crus 2) using the following formula: [(ROI − REF) / REF]. As described in Joling et al. (2018), we converted the standard REF mask from Montreal Neurological Institute (MNI)-space to subject space to be able to use a personalised REF for each patient. Furthermore, caudate/putamen ratios were calculated as described previously (Walker et al., 2004), using the following formula: [(left + right anterior caudate + nucleus accumbens binding) / (left + right posterior putamen binding)].

### 2.7 Voxel-based analysis

We also performed voxel-based analysis of covariance with age as covariate—since previous studies have shown natural ageing effects on ^123^I-FP-CIT binding both in striatal as well as extrastriatal brain areas (Koch et al., 2014; Varrone et al., 2013)—in SPM 12 to corroborate the findings of the significant results of the ROI analyses. Masks were used as previously reported (Joling et al., 2017). We used a statistical threshold of *P* < 0.050, Family-Wise Error (FWE) corrected for multiple comparisons.

### 2.8 Post-hoc analyses

We performed a post-hoc ROI analysis looking at 1) the midbrain, based on the results in Roselli et al. (2010), using an MNI mask warped to subject space, and 2) the volumes of the ROIs using FreeSurfer data.

### 2.9 Statistics

We assessed the distribution of the data by plotting histograms, examining Q-Q plots, and using Kolmogorov-Smirnov tests. Clinical variables were compared with either unpaired *t*-tests, or Mann–Whitney *U* tests, depending on normality. After checking for homogeneity of regression slopes between the variables *age* and *diagnosis*, and checking homogeneity of variance with Levene's test, we performed analysis of covariance between PD and DLB for mean binding ratio in each ROI with age as nuisance covariate.

To correct for multiple testing, we calculated corrected *P*-values (*P*_corr_) with Simple Interactive Statistical Analysis (SISA; http://www.quantitativeskills.com/sisa/calculations/bonhlp.htm), where the mean association between variables that are mutually correlated (binding ratios in six striatal ROIs and six extrastriatal ROIs) is used for the alpha correction (*r* = 0.766 striatal ROIs, *r* = 0.544 extrastriatal ROIs), and allows a less stringent correction than the Bonferroni method. For the striatal ROIs this resulted in a statistical threshold of *P*_corr_ < 0.033, and for extrastriatal ROIs *P*_corr_ < 0.022. We considered a *P*-value between *P*_*corr*_ and *P* = 0.050 for the binding ratios a trend. Effect sizes are reported as omega squared (*ω*^*2*^). We considered 0.01, 0.06, 0.14 as small, medium and large effect sizes, respectively (Kirk, 1996). All analyses were performed in SPSS 22 (IBM Corp, Armonk, NY, USA).

## 3 Results

### 3.1 Clinical characteristics

The clinical characteristics of both patient groups are summarized in Table 1. There was no significant difference in median disease duration (*P* = 0.831). For PD patients, the mean UPDRS-III was 29.44 (SD 13.32). Fifty DLB patients (94.3%) had one or more of the classical motor signs of parkinsonism registered in their patient records. Sixteen PD (30.2%) and 23 DLB (50.9%) patients had an MMSE-score below 25. ^123^I-FP-CIT SPECT scans were also rated in routine practice: forty-five (84.9%) DLB patients and 53 (100%) PD patients had an abnormal rated scan based on a combination of visual assessment and semi-quantitative analysis. Of the remaining eight DLB patients, two (3.7%) had a normal ^123^I-FP-CIT-SPECT scan, and in six (11.3%) the baseline scan was normal. All six subjects had had second scans at a later time-point, that were classified as abnormal.

**Table 1:** Patient characteristics.

	PD	DLB	Statistic/df/*P*
N	53	53	
Gender (f/m)	10/43	10/43	*χ*^2^ = 0.000/1/1.000
Age at ^123^I-FP-CIT SPECT scan, mean (SD)	69.50 (6.39)	67.83 (5.94)	*F* = 1.929/1,104/0.168
Disease duration, median (IQR)	3.00 (4.00)	3.00 (2.00)	*U* = 1239/102/0.831
MMSE, median (IQR)	26 (5)	24 (6)	*U* = 1166/105/0.130
UPDRS-III, mean (SD)	29.44 (13.32)	N/A^a^	
H&Y, median (IQR)	2 (0.5)	N/A	

SD, standard deviation.

PD, Parkinson's disease; DLB, Dementia with Lewy bodies; SPECT, single photon emission computed tomography; MMSE, mini mental state examination; UPDRS-III, Unified Parkinson's Disease Rating Scale–motor symptoms; H&Y, Hoehn & Yahr stage; IQR, inter quartile range; *U*, Mann-Whitney *U* test statistic; *χ*^2^, Chi squared test statistic.

a The presence of motor signs (bradykinesia, rigidity and tremor) was registered dichotomously.

### 3.2 ROI-based ^123^I-FP-CIT SPECT analyses

#### 3.2.1 Striatal DAT binding

**Fig. 2:**
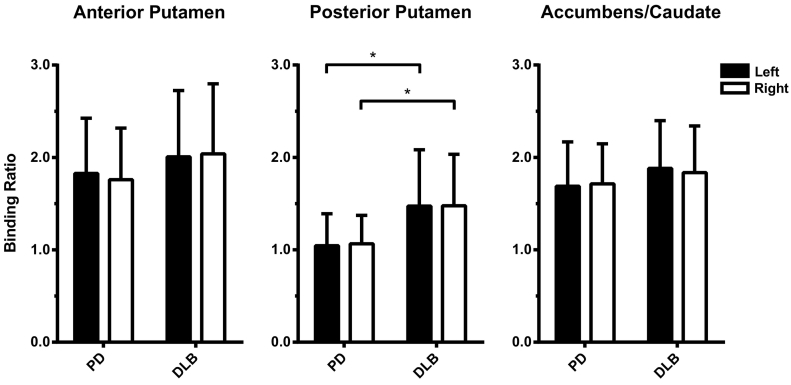
Mean striatal ^123^I-FP-CIT binding ratios in Parkinson's disease (PD) and dementia with Lewy bodies (DLB); error bars represent the standard deviation (SD); *P < 0.001.

Analysis of covariance with age as covariate showed significantly lower striatal ^123^I-FP-CIT binding in PD patients than in DLB patients in both left and right posterior putamen (left: *F*(1,103) = 18.363, *P* < 0.001, *ω*^*2*^ = 0.14; right: *F*(1,103) = 20.434, *P* < 0.001, *ω*^*2*^ = 0.15) (*P*_*corr*_ < 0.033). Caudate/putamen ratios were also significantly different, with a lower ratio in DLB than in PD (*U*(105) = 724.0, *P* < 0.001). There were no significant differences in ^123^I-FP-CIT binding between PD and DLB in the other striatal ROIs (Fig. 2). UPDRS-III scores in PD patients correlated negatively with ^123^I-FP-CIT binding in the bilateral anterior putamen (left: *r* = −0.296, *P* = 0.033; right: *r* = −0.414, *P* = 0.002), and trend-significantly the right posterior putamen (*r* = −0.285, *P* = 0.040), but not in the left posterior putamen (*r* = −0.208, *P* = 0.140) or the bilateral accumbens/anterior caudate (left: *r* = −0.178, *P* = 0.207; right: *r* = −0.254, *P* = 0.069). Analysing the striatal ROIs by most or least affected side (i.e., side with lowest versus highest binding ratio) did not differ significantly from the left versus right analysis (data not shown).

#### 3.2.2 Extrastriatal SERT binding

**Fig. 3:**
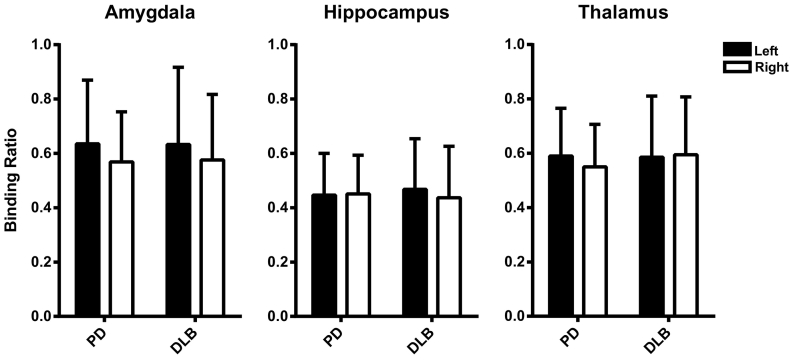
Mean extrastriatal ^123^I-FP-CIT binding ratios in Parkinson's disease (PD) and dementia with Lewy bodies (DLB); error bars represent the standard deviation (SD).

There were no significant differences in ^123^I-FP-CIT binding ratios between PD and DLB in the bilateral thalamus, amygdala and hippocampus. See Fig. 3.

### 3.3 Voxel-based ^123^I-FP-CIT SPECT analysis

**Fig. 4:**
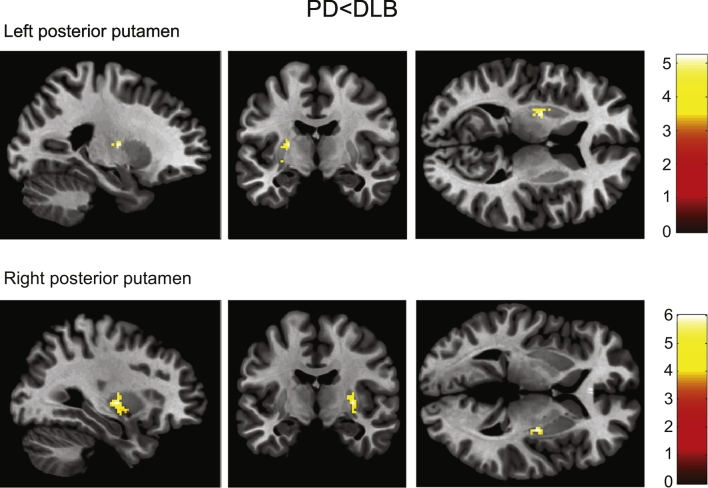
Voxel based analysis: striatal voxels in which Parkinson's disease (PD) patients has lower binding than dementia with Lewy bodies (DLB) patients, corrected for age, masked for posterior putamen.

Consistent with our ROI analysis, the voxel-based analysis showed significantly lower ^123^I-FP-CIT binding in the bilateral posterior putamen in PD compared with DLB, see Table 2 and Fig. 4. Also, the analysis confirmed the absence of significant differences between PD and DLB in the binding ratios in extrastriatal ROIs.

**Table 2:** Voxel-based analysis.

Region of interest	K_e_	*P*_*FWE*_ peak-voxel	*T*	x/y/z (mm)
Left posterior putamen	35	<0.001	5.22	−24/−8/10
	3	0.006	4.44	−30/−12/2
Right posterior putamen	70	<0.001	5.99	30/−8/2
	2	0.022	4.02	24/−2/12

Analysis on the ROIs with significant difference between patients with Parkinson's disease and dementia with Lewy bodies. K_e_, size of significant cluster of voxels; *P*_*FWE*_, Family-wise corrected *P*-value; *T*, T-statistic value; x/y/z, millimetres from the anterior commissure in Montreal Neurological Institute-space.

### 3.4 Post-hoc analysis

We did not find a significant difference between PD and DLB patients (*F*(1,103) = 0.397, *P* = 0.530) in the midbrain. In addition, we did not find a significant difference between PD and DLB in the volumes of the ROIs. Adding the ROI volumes as nuisance covariate in the ROI analysis of covariance did not alter the outcome substantially (data not shown).

## 4 Discussion

In this molecular imaging study we compared striatal and extrastriatal ^123^I-FP-CIT binding between DLB and PD patients matched for age and gender, with MMSE values in the same range of severity. As expected from the literature, we observed significantly lower ^123^I-FP-CIT binding in the bilateral posterior putamen in PD patients compared to DLB patients with a large effect size (left: *ω*^*2*^ = 0.14; right: *ω*^*2*^ = 0.15). This difference was observed in both ROI-based and voxel-based analyses. Contrary to our hypothesis, we did not find any significant group differences in ^123^I-FP-CIT binding in extrastriatal areas, including the thalamus, hippocampus, and amygdala.

The difference in striatal ^123^I-FP-CIT binding between PD and DLB corroborates the results of previous studies. O'Brien and co-workers, for example, reported a greater loss of ^123^I-FP-CIT binding in the posterior putamen in PD patients than in DLB patients, reflected by a flatter rostrocaudal gradient in DLB (O'Brien et al., 2004). The same group demonstrated that, in comparison to the DLB patients, the rate of decline in ^123^I-FP-CIT binding in the posterior putamen in PD patients was higher, measured over an interval of approximately one year (Colloby et al., 2005). Furthermore, other studies have also demonstrated that the posterior putamen in DLB patients is less affected than in PD patients (Piggott et al., 1999; Walker et al., 2004).

In our current sample, we observed no statistically significant differences in ^123^I-FP-CIT binding between PD and DLB in extrastriatal SERT-rich ROIs, including bilateral thalamus, amygdala and hippocampus. Since we did not include healthy age-matched controls, we cannot state with certainty that loss of SERT occurred in any of our patient groups, but loss of SERT in PD has been shown before with ^11^C‑3‑amino‑4‑(2‑dimethylaminomethylphenylsulfanyl)‑benzonitrile (^11^C-DASB), a SERT-selective positron emission tomography tracer (Politis et al., 2010). Furthermore, loss of serotonergic neurons has previously been observed in a neuropathological study of the dorsal and medial raphe nuclei in DLB patients (Benarroch et al., 2007); however, PD patients were not included in that study. In another histopathological study using SERT immunoreactivity that included both PD and DLB patients, dystrophic axons were found in the brainstem and hippocampus of both groups. Further quantitative morphometric analysis of SERT-positive neurons in the prefrontal cortex revealed significant differences between controls on the one hand and PD and DLB patients on the other hand, but not between the two diseases (Azmitia and Nixon, 2008). Moreover, the prevalence of neuropsychiatric symptoms associated with serotonergic deficits, like anxiety, is not consistently different between PD and DLB (Borroni et al., 2008; Chiu et al., 2016; Kao et al., 2009). Taken together, the results of previous studies and our observations converge to suggest that SERT-presenting neurons degenerate both in PD and DLB, and that the severity of loss of these neurons is not necessarily different between diseases.

In a small-size (15 PD and 16 DLB patients) molecular imaging study, a profound loss of ^123^I-FP-CIT SERT binding in the midbrain was reported in DLB patients compared with PD (Roselli et al., 2010). Because of this discrepancy with the results of our present study involving other extrastriatal brain areas, we performed an additional post-hoc analysis of midbrain ^123^I-FP-CIT SERT binding and failed to find a between-group difference for the midbrain. The discrepancies between the two studies may be explained by the choice of the occipital cortex as a reference region, whereas we used the cerebellum, which is relatively devoid of SERT (Kish et al., 2005). Since the occipital cortex does contain some serotonergic terminals (Kish et al., 2005), using the occipital cortex as a reference region may result in an underestimation of the specific-to-nonspecific binding ratios. Furthermore, in our study SRI users were excluded, whereas in the Roselli study SRIs were withheld for seven days prior to scanning. Since some SRIs have a half-life of up to 6 days, and pharmacokinetics are often slower in the elderly (Marken and Munro, 2000), these drugs may still have occupied the SERT at the time the scans were performed. Lastly, we chose not to include the midbrain in our primary ROI-based analyses, since it is a region that also contains dopaminergic neurons and is closely adjacent to other ROIs such as the thalamus, with the risk of cross contamination by activity in these regions.

Within our DLB group there was some heterogeneity in phenotype: we observed that two clinically diagnosed DLB patients had markedly higher ^123^I-FP-CIT binding ratios for DAT and SERT than the other DLB patients. Repeating the analyses without these two cases, did not significantly change the results of the present study (data not shown). Normal-rated ^123^I-FP-CIT SPECT scans have been reported in approximately 10% of DLB patients (Thomas et al., 2017; van der Zande et al., 2016). In some of these cases, ^123^I-FP-CIT SPECT binding becomes abnormal later in the disease process (van der Zande et al., 2016). So, such cases may represent a subgroup of DLB patients in whom degeneration in cortical brain areas is initially more severe than in subcortical brain areas (cortical subtype of DLB) (van der Zande et al., 2016). We did not exclude these patients from our analyses, because striatal ^123^I-FP-CIT binding in this subgroup of DLB patients is known to decline (Colloby et al., 2005). This is something we also saw in 6 cases, where the first ^123^I-FP-CIT SPECT scans were normal, and later scans abnormal. There was no difference in age, disease duration or MMSE scores in this subgroup compared with the rest of the DLB patients.

A strength of the present study is the subject-based anatomical ROI approach that was used, as opposed to the standardised ROIs used in other studies. Another strength is the relatively large sample size. A relative limitation is the availability of a single ^123^I-FP-CIT SPECT scan per subject: we previously showed that the optimal time point for assessment of SERT binding is around 2 h after injection (Booij et al., 2007), therefore ideally we would have acquired ^123^I-FP-CIT SPECT scans at two time points after injection. Due to the retrospective nature of this study and the use of clinical scans this was not possible. Furthermore, we know from our previous study on SERT binding that ratios of specific to non-specific ^123^I-FP-CIT binding to SERT remain stable up to 3 h post-injection, Therefore, relative differences in ^123^I-FP-CIT binding to SERT are still detectable at 3 h post-injection.

In this study, patients on SRIs were excluded, since these drugs block the SERT, and consequently may influence ^123^I-FP-CIT binding to the SERT. However, inevitably in this retrospective study performed in routine care patients, many were using common dopaminergic agents (e.g., levodopa or dopamine agonists). Although these classes of drugs are usually allowed when performing ^123^I-FP-CIT SPECT in routine patient care (Booij and Kemp, 2008), we cannot exclude that the use of these drugs may have had an impact on the results of the present study. However, if such an effect indeed occurred, one would expect this to impact DAT binding in all subregions of the striatum. Consequently, we believe it is unlikely that such an effect would explain the difference in ^123^I-FP-CIT binding between PD and DLB patients.

Another discussion point in our study is that we included patients from two different outpatient clinics, which introduced the risk of a diagnostically different view on equivocal cases. Moreover, there was no consistency in the assessment of motor- and neuropsychiatric variables, both representing important symptom groups in PD and DLB. Since UPDRS-III scores were not determined for the DLB patients we could not compare neural correlates of motor function, which are usually linked to the loss of striatal dopaminergic synapses, also in DLB (Del Sole et al., 2015). Therefore, we cannot exclude the possibility that we failed to find lower caudate ^123^I-FP-CIT binding in DLB than in PD, and conversely found lower ^123^I-FP-CIT binding in the posterior putamen in PD just because PD patients had a more severe parkinsonism. A possible mitigating factor for this limitation could be a similar disease duration.

This is another potential limitation of our study: We did not use disease duration as an inclusion criterion. Disease duration was, however, fortunately not different in both diseases. Although we defined disease duration for DLB and PD differently, which by definition makes it less suitable as inclusion criterion: for DLB patients we chose the first appearance of cognitive symptoms as an approximation of disease onset, whereas for the PD patients we used the patient-reported onset of the cardinal motor symptoms.

In conclusion, this study is the first to extensively compare ^123^I-FP-CIT binding in both striatal and extrastriatal brain areas between PD patients and DLB patients in a relatively large cohort. The results confirm earlier observations of a more severe loss of ^123^I-FP-CIT binding in the posterior putamen in PD patients than in DLB patients. Furthermore, we found no differences in extrastriatal ^123^I-FP-CIT binding between the two disease groups, which suggest similar degree of degeneration of serotonergic structures in both diseases.

## Disclosure statement

MJ: salary was paid by a research grant from GE healthcare (paid to the institution). JB: received research grants from GE Healthcare (paid to the institution). OAVDH: is co-applicant of research grants obtained from GE Healthcare (paid to the institution). HWB: is co-applicant of research grants obtained from GE Healthcare (paid to the institution) CV, JJZ, AWL: declare no conflicts of interest.
